# How linked are national HIV and SRHR strategies? A review of SRHR and HIV strategies in 60 countries

**DOI:** 10.1093/heapol/czw119

**Published:** 2017-11-24

**Authors:** Jonathan Hopkins, Lynn Collins

**Affiliations:** 1 IPPF, 4 Newhams Row, London SE1 3UZ, UK; 2 UNFPA, 605 Third Avenue, New York, NY, USA

**Keywords:** Sexual health, reproductive health, human rights, HIV, integration, sexually transmitted infections, condoms

## Abstract

The connection between HIV and sexual and reproductive health and rights (SRHR) is widely recognised along with the benefits of linking them at the legal/policy, health systems, and service delivery levels. However, despite increased rhetoric about the need for this three-tiered approach, integrated service delivery has not been fully addressed at the legal/policy level through national strategies. Thus a review of HIV and SRHR strategies was conducted for 60 countries, determining the extent to which they reflected the intersections between HIV and SRHR. Each HIV strategy was scored on whether five key SRHR components were incorporated and had an associated measurable target. SRHR strategies were similarly assessed for incorporation of five HIV components and associated targets. HIV strategies had a higher level of inclusion of SRHR components with a global average of 6.6/10 compared to 3.7/10 for SRHR strategies. The highest scoring component was the elimination of mother-to-child transmission of HIV (EMTCT) and the lowest was SRHR of people living with HIV. Countries with higher scores in one strategy tended to have higher scores in the other but there was no difference over time. Whilst there has been increased global commitment since 2004 to link SRHR and HIV, insufficient headway has been made in linking related national strategies. Although EMTCT is included with targets in the majority of HIV and SRHR strategies, the broader SRHR needs of women living with HIV are not. Also, condoms are not being considered an effective triple protection tool. HIV and SRHR strategies provide direction and targets which ultimately may influence funding and vice versa. Therefore, it is essential that these strategies are right-based and incorporate the key connections between SRHR and HIV with measurable targets to realise the full benefits of a joint response.

**Key Messages**

- While there has been increased commitment over the past 12 years to better link HIV and sexual and reproductive health and rights (SRHR) by exploiting their intrinsic connections, insufficient headway has been made at the strategy level.
- Specific areas that need intensified focus include the full scope of SRHR needs of women living with HIV (beyond the elimination of mother to child transmission of HIV) and the promotion of condoms’ effectiveness for triple protection against HIV, other STIs and unintended pregnancies.
- More comprehensive and resilient health systems require integrated service delivery to be ‘normalised’ through the systematic inclusion of key integration components in both HIV and SRHR strategies.

## Introduction

The intrinsic connections between HIV and sexual and reproductive health and rights (SRHR) are well-established, especially as HIV is predominantly sexually transmitted or associated with pregnancy, childbirth and breastfeeding (UNFPA *et al*. 2013). Similarly sexually transmitted infections (STIs) can increase the risk of HIV acquisition and transmission (WHO 2007). People living with HIV have specific SRHR needs including but not limited to the prevention of mother-to-child transmission of HIV, which requires an integrated service delivery response (Brickley *et al.* 2011). Linkages between SRHR and HIV lead to a number of important health and well-being benefits (WHO *et al.* 2005; Kennedy *et al.* 2010). For example, integrating HIV into SRH services can lead to better HIV testing outcomes (Church *et al.* 2013a), more consistent condom use (Church *et al.* 2014), improved quality of care (Mutemwa *et al.* in this supplement), potential for better use of scarce human resources for health (Obure *et al.* 2015; Integra Initiative 2015), and potential for reduced HIV-related stigma and discrimination (Church *et al.* 2013b; Colombini *et al.* 2014). SRHR and HIV linkages may also improve coverage, access to, and uptake of both SRHR and HIV services for at risk/vulnerable and key populations including people living with HIV, men who have sex with men, sex workers, people who inject drugs, and transgender people (Kennedy *et al.* 2010).

**Figure 1.**
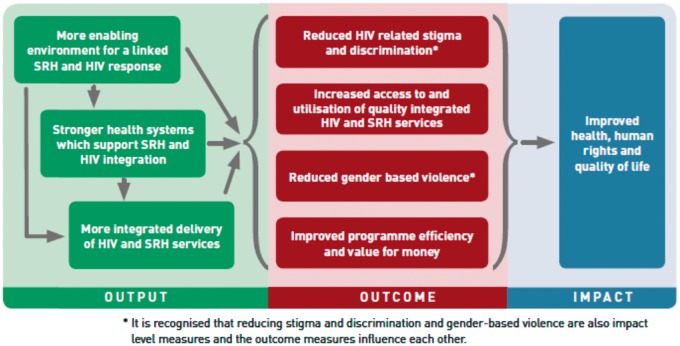
Theory of change for SRH and HIV linkages

Linkages and integration have various definitions. For the purposes of this paper, the definitions used are those agreed by key organisations working on the HIV and SRHR linkages agenda. Linkages refer to ‘the bi-directional synergies in policy, programmes, services and advocacy between SRHR and HIV. It refers to a broader human rights-based approach, of which service integration is a subset’ (IPPF *et al.* 2009). Integration refers to ‘the service delivery level and can be understood as joining operational programmes to ensure effective outcomes through many modalities (multi-tasked providers, referral, one-stop shop services under one roof, etc.)’ (IAWG on SRH and HIV Linkages *et al.* forthcoming). An important distinction in this terminology is that integration focuses on the service delivery level and linkages is broader, also including health systems and the enabling environment (legal/policy). The interactions between these levels have been articulated in a Theory of Change (Figure 1, IPPF *et al*. 2013) .

As the Theory of Change shows, service integration alone is not sufficient for the full benefits of SRHR and HIV linkages to be realised. A holistic approach is needed that also impacts on the health systems and enabling environment of which HIV and SRH-related policies and strategies form a key component (IPPF *et al*. 2009).

The policy rationale for linking HIV and SRHR has been articulated since the early 1990s (Church and Mayhew 2009). A defining moment was the International Conference on Population and Development (ICPD) held in Cairo in 1994 (Gruskin *et al.* 2007; Church and Mayhew 2009; Wilcher *et al.* 2009; Hope *et al.* 2014). The ICPD Programme for Action clearly set out the need for integrated service provision within a primary healthcare approach (Para 8.17, UNFPA 2014) and recommends the integration of HIV national plans and strategies with population and development strategies (Para 8.30, UNFPA 2014).

In the early 2000s, the swift rise in HIV funding through the US President’s Emergency Plan for AIDS Relief (PEPFAR) and the Global Fund to Fight AIDS, TB and Malaria (Global Fund), both of which included restrictions excluding funding non-condom contraception, initially increased verticalisation of the HIV response (Gruskin *et al.* 2007; Wilcher *et al.* 2009). However in 2004, marked recognition of the importance of linking SRHR and HIV, with major international organisations issuing statements calling for stronger linkages between SRHR and HIV. For example, *The Glion Call to Action on Family Planning and HIV/AIDS in Women and Children* (WHO 2007) was built on the understanding that preventing/eliminating mother-to-child transmission of HIV (PMTCT/EMTCT) requires delivery of interventions through joint SRH and HIV services. *The New York Call to Commitment; Linking HIV/AIDS and Sexual and Reproductive Health* went even further, beyond EMTCT, addressing all aspects of a linked SRHR and HIV response. These, and a number of agreements and strategies that followed, acknowledged the importance of SRHR and HIV Linkages to meeting the Millennium Development Goals (MDGs) and later the Sustainable Development Goals (SDGs) (UNAIDS 2005; African Union 2006; Wilcher *et al.* 2009; Global Fund 2011; United Nations 2011; UNAIDS 2011, Hope *et al.* 2014; UNAIDS 2015a). This growing political will also be seen at the intergovernmental level. For example, in 2015 the Southern Africa Development Community (SADC) launched the *Minimum Standards for the integration of HIV and Sexual and Reproductive Health* which seek to promote and support efforts by Member States to better integrate SRHR and HIV into national policies and frameworks (Southern African Development Community 2015).

High level political will for SRHR and HIV Linkages, however, does not necessarily translate to national level action at the legal/policy, health systems and service delivery levels. Smit *et al.* (2012) found that ‘policy directives mandating the delivery of healthcare in an integrated fashion are needed to normalise integration as a requirement, rather than an optional extra.’ The focus, therefore, needs to be on actions across the legal/policy, health systems, *and* service delivery levels. A review of the findings of the first 20 countries to undertake the Rapid Assessment Tool for SRH and HIV Linkages (IPPF *et al.* 2009) found that one of the most common gaps was at the legal/policy level with a lack of incorporation of HIV and SRHR jointly in national policies (Lusti-Narasimhan *et al.* 2014). However, very little data exist on how well SRHR and HIV strategies are linked. With this in mind, as part of a process to develop an SRHR and HIV Composite Index of Linkages Indicators, analysis was undertaken of the national HIV strategies and SRHR strategies in 60 countries.

## Methods

To better understand the extent to which SRHR and HIV strategies were linked, a review of current HIV strategies and SRHR strategies was conducted for 60 countries – see Table 1. The dataset was created as part of a process to develop a composite SRH and HIV Linkages Index of indicators led by UNFPA, IPPF and WHO with the support of an expert panel (UNFPA *et al.* forthcoming). The countries were chosen for the Index using the following criteria:

**Table 1.** List of countries chosen for the composite SRH and HIV Linkages Index (by region)

Eastern & Southern Africa	West & Central Africa	Middle East & North Africa	Asia & the Pacific	Eastern Europe and Central Asia	Latin America & the Caribbean
Angola	Benin	Lebanon	Afghanistan	Kyrgyzstan	Barbados
Botswana	Burkina Faso	Morocco	Papua New Guinea	Russian Federation	Belize
Burundi	Cameroon	Sudan	Bangladesh	Ukraine	Dominican Republic
Eritrea	Central African Republic	Tunisia	Cambodia		Guatemala
Ethiopia	Chad		China		Guyana
Kenya	Congo, Dem. Rep		India		Haiti
Lesotho	Cote D'Ivoire		Indonesia		Jamaica
Malawi	Ghana		Maldives		
Mozambique	Guinea Bissau		Myanmar		
Namibia	Mali		Nepal		
Rwanda	Niger		Pakistan		
South Africa	Nigeria		Philippines		
South Sudan	Senegal		Sri Lanka		
Swaziland	Togo		Viet Nam		
Tanzania					
Uganda					
Zambia					
Zimbabwe

- The country had shown previous interest in SRHR and HIV Linkages as they had completed a Rapid Assessment using the Rapid Assessment Tool for SRH and HIV Linkages (IPPF *et al.* 2009; IAWG for SRH and HIV Linkages 2014);
- The country was a priority country for one of the following multilateral donors/agencies: The Global Fund to Fight AIDS, TB and Malaria; PEPFAR; or UNFPA (as part of a Memorandum of Understanding between UNFPA and the Global Fund).

There was considerable overlap between the different categories and led to a total of 60 countries being selected.

The coding was undertaken between April and June 2015. For each country selected, a search was conducted for the current national HIV strategy and the current national SRHR strategy. For HIV strategies, National HIV Strategic Frameworks or National HIV Strategic Plans were sourced through an internet search and direct contact with the country offices of UNFPA and Member Associations of the International Planned Parenthood Federation (IPPF). The majority of HIV strategies were five years and the most recent HIV strategy was used provided it was still in effect in 2014 or later. For SRHR strategies a similar search strategy was utilised but this had to be refined as most countries do not have a stand-alone SRHR strategy. SRHR is included in a range of different health sector documents including: Maternal and Infant Health Plans; Millennium Development Goal Acceleration Frameworks that focused on goal 5 – maternal mortality; and National Health Plans (looking at SRHR components). The website National Planning Cycles (www.nationalplanningcycles.org) was used as a key source for finding relevant strategies and also direct contact with UNFPA country offices and IPPF Member Associations. The first priority was to find the national SRHR/SRH/RH strategy or policy, but where these did not exist, the other aforementioned documents were used (in order of priority listed above). As many of these strategies and plans did not have a cut-off date, a ten year cut-off was adhered to, meaning that no document dated before 2005 was used in the dataset.

Each of the strategies was reviewed to see whether selected linkage components had been included as priorities. The components were determined based on a review of the HIV and SRHR linkages literature which articulates intrinsic connections between SRHR and HIV (WHO *et al.* 2005; UNAIDS 2010a; IAWG on SRH and HIV Linkages *et al.* forthcoming) and were endorsed by the IAWG on SRH and HIV Linkages. The selected linkage components are:

- Elimination of mother-to-child transmission of HIV (EMTCT): uses a four pronged approach to preventing HIV in pregnant women, mothers and their children and keeping mothers alive, delivered through the SRHR platform (UNAIDS 2011)
- Sexually transmitted infections (STIs): HIV is primarily sexually transmitted, and non-HIV STIs can increase HIV acquisition and transmission (WHO 2007)
- Condoms: confer triple protection from HIV, other STIs and unintended pregnancies
- Sexual and reproductive health (SRH) of people living with HIV (PLHIV): SRH must be tailored to meet the needs of PLHIV including rights, see below for the full scope of components (GNP+ & UNAIDS 2013)
- Gender based violence (GBV): a key component of SRHR and a cause and consequence of HIV (UN Commission on the Status of Women 2013)
- HIV testing and counselling (HTC): relevant to SRHR since HIV is predominantly sexually transmitted and associated with maternal health and family planning through mother-to-child transmission of HIV (UNFPA *et al*. 2013)

For the HIV strategies and SRHR strategies, five of the above six linkage components were assessed - see Table 2.

**Table 2.** Linkage components assessed in HIV strategies and SRHR strategies

Linkage component	HIV strategies	SRHR strategies
**EMTCT**	EMTCT	EMTCT
**STIs**	STIs other than HIV	STIs (with reference to HIV)
**Condoms**	Condoms (with reference to STI/pregnancy prevention)	Condoms (with reference to HIV prevention)
**SRH of PLHIV**	SRH of PLHIV	SRH of PLHIV
**GBV**	GBV	N/A*
**HTC**	N/A**	HTC

* GBV is one of the core components of SRHR outlined in ICPD (UNFPA 2008) so it is assumed that GBV is included in SRHR strategies.

** HTC is a standard part of HIV services so is assumed to be included in HIV strategies.

Each document was reviewed and each linkage component was given a score out of 2 using a categorised rating system. A score of 1 meant there was a mention of this specific issue but no measurable target and a score of 2 meant the component was both mentioned and included a measurable target. A measurable target was defined as the inclusion of numbers, percentages or words indicating a specific target or goal to be reached. Examples of targets or goals include: a 50% increase in STI services by 2018; reducing mother-to-child transmission of HIV to < 2%; 10 million condoms distributed annually to reduce transmission of HIV; or all family planning clinics to provide HTC services. ‘Services that support the SRH of PLHIV should improve’ was not sufficient for this component to be given a score of 2. Thus the maximum score for each strategy was 10. The scores were initially given by a designated researcher and then the whole dataset was fully reviewed by two experts in SRHR and HIV linkages to validate the scores.

Unlike the other linkage components, SRH of PLHIV was often not explicitly stated in this way in the HIV and SRHR strategies. Therefore a more detailed scoring criteria was developed for this component with a search conducted in each strategy for the any of the following specific SRH services for PLHIV: prevention and treatment of STIs, including viral hepatitis; counselling and support for a satisfying sex life, including but not limited to improving libido and treating sexual dysfunction; family planning, including infertility and contraceptive services; cervical, breast and other related cancer screening and management; and access to appropriate, safe and non-coerced termination services. If any one of these services was mentioned for PLHIV then a score of 1 was given, and if measurable target was included then this scored 2.

As part of the analysis, in order to better understand some of the factors potentially influencing the linkage scores, three variables were selected:

- The relationship between the scores for HIV and SRHR strategies within a country – it is expected that a higher score in one strategy will also be reflected in a higher corresponding score in the other because the connections between SRHR and HIV are recognised at the national level.
- Changes in score over time – since global political commitment to HIV and SRHR linkages has intensified since 2004, this should increasingly influence to various extents the degree of linkage within newer national strategies.
- Regional variation – there would potentially be some similarities based on common health needs in neighbouring countries and also the potential influence of regional bodies (e.g. Southern Africa Development Community).

## Results

**Figure 2.**
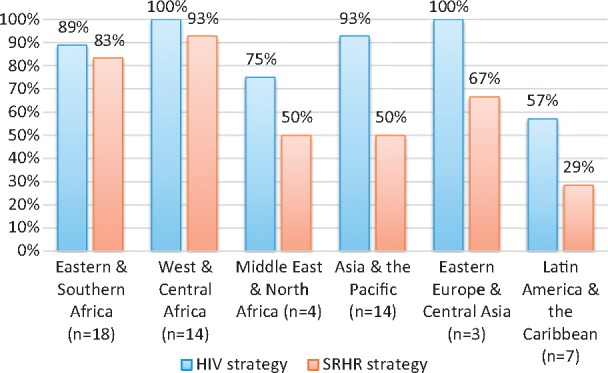
Strategy availability by region

HIV strategies were found for 53 countries (88.3%) and SRHR strategies for 41 countries (68.3%). Strategies were located for every region but availability varied and SRHR strategies were consistently less available (see Figure 2).

**Figure 3.**
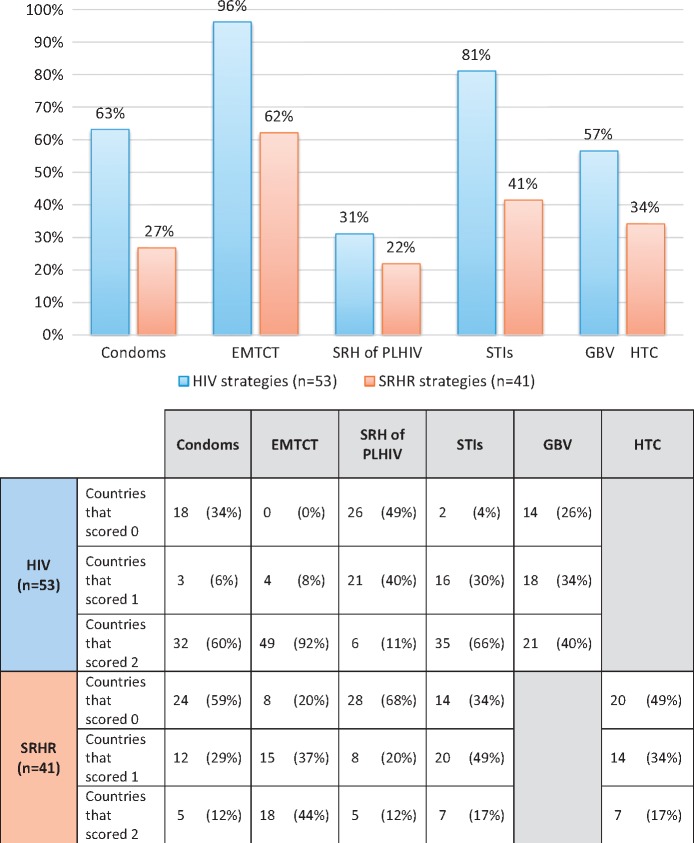
Component scores: Percent of maximum score across dataset and score breakdown

*Linkages Scores:* The maximum possible score for each strategy – being scored against whether five linkage components had been included as priorities – was 10. The range of scores for reviewed HIV strategies was between 3 and 10 and for SRHR strategies was between 0 and 10. Of the 53 HIV strategies found, the average score was 6.6/10 and for the 41 SRHR strategies the average score was 3.7/10. Figure 3 shows the percentage of strategies achieving the maximum score by the component and the breakdown of the scores.

For HIV strategies, EMTCT scored the highest receiving 96% of the maximum score across the 53 countries in the dataset (a component would score 100% if every country scored a maximum 2). Every country scored at least 1 on this measure and 49 countries (92%) also included a measurable target. STIs also scored second highest achieving 81% of the total score and all but two countries mentioned STIs other than HIV in their strategy. However, only 35 countries (66%) had a measurable target. Condoms (with reference to STI prevention or contraception) scored 63%. 32 strategies (60%) had a measureable target thus scoring 2 and three strategies (6%) scored 1. The fourth highest was GBV which received 57% of the total score. Fewer strategies scored 0 on GBV compared to condoms – 14 rather than 18 – but the lower overall score was due to fewer strategies scoring the maximum by including a related measurable target – 21 rather than 32 for condoms. The SRH of PLHIV scored the least achieving just 31% of the maximum score. Twenty-six strategies (49%) did not mention it and only six strategies (11%) included a measurable target.

For SRHR strategies, the scores were lower than for HIV strategies in every area but the order was broadly similar. The component which scored the highest was EMTCT with 62% of the maximum score. Fifteen strategies (37%) scored 1 and a further 18 strategies (44%) scored 2. Second was STIs with reference to HIV which scored 41%. 20 strategies (49%) scored 1 and seven strategies (17%) scored 2. HTC was next with a score of 34% of the maximum. Twenty strategies (49%) scored 0, 14 (34%) scored 1 and seven (17%) scored 2. Condoms for HIV prevention scored 27% of the maximum. Twenty-four strategies (59%) scored 0, 12 strategies (29%) scored 1 and five strategies (12%) scored 2. Finally, the SRH of PLHIV was the lowest score – as for the HIV strategies – with a score of 22% of the maximum. Twenty-eight strategies (68%) scored 0, eight strategies (20%) scored 1, and five (12%) scored 2.

### Possible factors affecting linkage scores

The results of the analysis of the three selected variables potentially affecting linkage scores are as follows:

### i) Relationship between strategy scores

**Figure 4.**
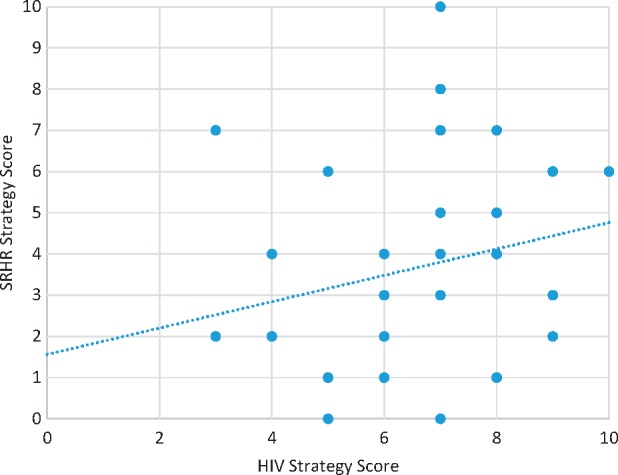
Relationship between HIV strategy and SRHR strategy scores

Whilst the SRHR strategies scored lower than the HIV strategies, there was a positive association between them in the 41 countries where there were data for both an SRHR strategy and an HIV strategy (see Figure 4).

### ii) Differences over time

When comparing scores against the year that each strategy began scores did not increase over time for either the HIV or the SRHR strategies.

### iii) Regional differences between scores

**Figure 5.**
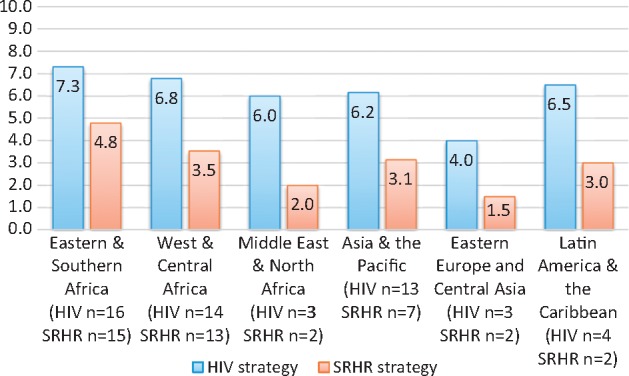
Scores (/10) by region

As Figure 5 shows, the two regions that scored the highest for both SRHR and HIV strategies are Eastern and Southern Africa and West and Central Africa. The next three regions have very similar scores for HIV in Latin America and the Caribbean (6.5), Asia & the Pacific (6.2) and the Middle East and North Africa (6.0) for HIV. Eastern Europe and Central Asia scored the lowest with an average score of 4.0 across the three countries with HIV-strategies. For SRHR strategies, scores were lower than for the HIV strategies in every region. Eastern and Southern African countries still scored the highest with an average score of 4.8 with West and Central African countries scoring 3.5. Countries in Asia and the Pacific and Latin America and the Caribbean scored 3.1 and 3.0 respectively with countries in the Middle East and North Africa scoring 2.0 and Eastern Europe and Central Asia scoring and average of 1.5 in the two countries that had an SRHR strategy. It is noted that the sample size is very small in the Middle East and North Africa, Eastern Europe and Central Asia, and Latin America and the Caribbean.

## Discussion

### Limitations

Of the 60 countries in the sample, HIV strategies were not found for seven countries (12%) and SRHR strategies were not found for 19 countries (32%). The reasons for this varied and included absence or expiration of a strategy and inability to locate it within the research timeframe. Moreover, since dedicated SRHR strategies do not exist for many countries some SRHR strategies used were components of a broader health strategy. The scoring system has a subjective element to it so to minimise bias of any one reviewer, the full dataset – after being coded by a designated researcher – was reviewed by two SRHR and HIV linkages experts. The linkage components were selected as proxies and do not represent the entirety of SRHR and HIV components. For example EMTCT has four elements, however, there was no requirement for all the elements to be explicitly mentioned when scoring the strategies. Finally, some strategies noted the importance of integration as a concept and means of implementation but this is not necessarily reflected in the score which was assessing whether select SRHR and HIV components had been included as priorities.

### Summary of findings and implications

Despite the plethora of international agreements and intensified commitment to strengthen SRHR and HIV linkages, the varying extent to which HIV and SRHR strategies incorporated the linkage components shows that this commitment is not consistently given priority in relevant health sector strategies. The bi-directional incorporation of the key overlapping components of SRHR and HIV is weak – particularly in SRHR strategies which have a consistently lower score on each linkage component than HIV strategies. Several factors and influences may account for this: the wide scope of SRHR, yet the narrow scope of some of the SRHR strategies available for review; less engagement of HIV experts in SRHR spheres; and greater rights-based advocacy within the HIV-response which has included calls for SRHR to be included.

There has been considerable debate as to why there has not been sufficient progress overall in linking SRHR and HIV at the national level (IPPF *et al*. 2010). Structurally often HIV and SRHR departments are separate and joint planning and coordination do not take place – including in developing new HIV and SRHR strategies and ultimately in delivering services, exacerbated by vertical systems for commodities, logistics and monitoring and evaluation (Lusti-Narasimhan *et al.* 2014). Funding – especially from external donors – continues to be a hindrance since it tends to be allocated through existing vertical structures with stringent conditions for how these funds will be spent. A lack of coordination between donors and also competition at the national level to access scare resources available for health contributes to verticality. Additionally, there continues to be uneasiness within the SRHR community to take on additional controversial areas such as sexual rights and decriminalisation of sex work. Despite the global advocacy for SRHR and HIV linkages, there is still a lack of understanding of how these two areas are actually connected.

Nevertheless, the varying results of the study by component bear different implications for the suggested next steps.

### Elimination of mother to child transmission (EMTCT)

Elimination of Mother to Child Transmission (EMTCT) was the most successful – highest scoring – component for both the HIV and SRHR strategies. The reason for this high score is likely due in part to the high profile focus of the prevention of mother-to-child transmission from powerful global actors. In 2011 the UN held a Global Assembly Special Session resulting in the UNGASS Declaration of Commitment (United Nations 2001), which was then given further impetus with the commitments from Prevention of Mother-to-Child Transmission High Level Global Partners Forum in 2005 (Global Partners Forum 2005; IATT on Prevention of HIV Infection in Pregnant Women, Mothers and their Children 2007) and then the *Global plan towards elimination of new HIV infections among children by 2015 and keeping their mothers alive* (UNAIDS 2010b) which started in 2011. EMTCT requires an integrated approach through HIV and SRHR platforms as it is focused on interventions affecting pregnancy, childbirth and breastfeeding, unintended pregnancies, STIs, and GBV, so *de facto* requires integrated SRHR and HIV services in order to deliver it.

### Sexually transmitted infections (STIs)

HIV is predominantly a sexually transmitted infection and when HIV was incorporated in SRHR strategies this was generally the context within which it was viewed. The conclusive evidence that the presence of non HIV ulcerative and inflammatory STIs, such as gonorrhoea, chlamydia and syphilis, can increase the likelihood of HIV acquisition and transmission (Grosskurth *et al.* 1995) was a key factor in driving global policy recognition of the need to treat STIs as a HIV prevention measure in the mid-1990s and twenty years later has been recognised in 51 out of 53 HIV strategies. However there is certainly room for improvement particularly in terms of targets which appeared in only 27 HIV strategies and just 7 SRHR strategies. Targets require resources to achieve and therefore real government commitment. The lack of STI targets in the SRH strategies is likely to be indicative of the continuing siloed donor response whereby STI treatment is seen more as a ‘sexual health’ issue allied to HIV and not the remit of ‘reproductive health’. An exception is the recent guidance on validating dual elimination of mother to child transmission of HIV and syphilis (WHO 2014).

### Condoms

Condoms are currently the only effective multipurpose prevention technology that, when used consistently and correctly, protect against HIV, other STIs and unintended pregnancy. Given that condom use can meet a number of different health-related targets in both the HIV and SRHR spheres it would be expected that this multipurpose nature of condoms would be recognised. However, the findings of the study show that the maximum benefits of condoms are frequently not being recognised in the strategies – again largely because of siloed responses in which ‘HIV condoms’ and ‘condoms for family planning’ are understood, procured and dispensed separately, also family planning strategies tend to favour long-term acting reversible methods (Stanback *et al.* 2015). Given that both sets of strategies are employing the same biomedical product, coordinating responses are essential, beginning with noting the multi-functionality of condoms.

### SRH of PLHIV

Whilst activities to support EMTCT are incorporated and have targets in both HIV and SRHR strategies – especially HIV testing and treatment in antenatal settings – the availability of broad-scoped integrated SRHR and HIV services for PLHIV is still limited and human rights violations are still occurring (Salamander 2014; Loutfy *et al.* 2015). GNP+ and UNAIDS in the seminal publication ‘Positive Health, Dignity and Prevention: Operational Guidelines’ state:

‘Sexual and reproductive health and rights must be recognised and exercised by everyone regardless of his or her HIV status. Following diagnosis, people living with HIV continue to have the same needs and desires for intimacy, sexual activity, family, and community as before. Positive Health, Dignity and Prevention aims to create the conditions for people living with HIV and their sexual partners to be free to make informed choices regarding whether and how to be sexually active and fulfilled and whether, when and how to conceive and enjoy a family’ (p13, GNP+ and UNAIDS 2013)

Despite this, as the findings have shown, HIV and SRHR strategies are virtually silent on the broader SRHR needs of people living with HIV with this being the worst performing area for both HIV and SRHR strategies. Renewed effort is needed to advocate for stigma-free rights-based SRHR services for people living with HIV – including: pre-conception; infertility; contraception (non-coercive and full range); cervical, breast and other related cancer screening and management; prevention and treatment of STIs including viral hepatitis and syphilis, counselling and support for a satisfying sex life; access to appropriate, safe and non-coercive termination services (where legal). Such SRHR services need to be based on recognition of the rights of people living with HIV such as the right to make fertility decisions and live free of violence including coerced/forced sterilisation.

### Gender-based violence

The links between GBV and HIV are bidirectional with GBV both a cause and a consequence of HIV (UN Commission on the Status of Women 2013). Intimate partner violence has been shown to increase the risk of HIV infection by around 50% and violence (and the fear of violence) may deter women and girls from seeking HIV testing, disclosing their HIV status, and seeking other services for their HIV and SRHR needs (Kouyoumdjian *et al.* 2003; WHO and UNAIDS 2013). Given this dual link it would be expected that HIV strategies would include gender-based violence and whilst nearly three quarters of strategies (39) mention it, only half (21) include a measurable target. Much of the focus in HIV programming is on biomedical approaches, sometimes at the expense of addressing behavioural and structural aspects which are required to respond to GBV. This is a missed opportunity for HIV-strategies to support this key area of SRHR which also has significant consequences for HIV, including for key populations such as men who have sex with men, sex workers, people who inject drugs and transgender people.

### HIV testing and counselling

A key component of the new UNAIDS 2016-2021 Fast Track Strategy (UNAIDS 2015a) is the 90-90-90 target by 2020: 90% of people living with HIV know their status, 90% of those receive treatment and 90% of those on treatment are virally suppressed. In order to achieve the first ‘90’, every opportunity needs to be taken to increase access to HIV testing that is confidential, correct, and includes counselling, consent, and connection to relevant follow-up services (WHO 2015). However these links are not currently being made in SRHR strategies. Research shows that people with greater exposure to family planning or post-natal care facilities that had integrated HIV testing and counselling were more likely to know their HIV status (Church *et al.* 2013a). Therefore, this is a key area of focus to achieve the 90:90:90 treatment targets.

### Possible factors affecting linkages between HIV and SRHR strategies

The positive association in scores for HIV and SRHR strategies is to be expected. When an HIV strategy more fully incorporates SRHR components, the SRHR strategy in the same country tends to more fully incorporate HIV, and vice versa. This could be due to recognition at the highest levels of government of the interconnection between HIV and SRHR, which may decrease the level of verticality between the HIV and SRHR programmes. The importance of improved coordination between these two traditionally ‘siloed’ sectors has been shown in other studies to be a key driver of improved integration (IPPF *et al.* 2009), however further research is needed in this area.

However, some countries are still lagging behind in increasing global political commitment for SRHR and HIV Linkages – including through global member state agreements at the UN such as the 2011 UNGASS agreement – is not fully translating to more interconnected national strategies for HIV and SRHR. Given the increasing number of high level commitments at the Global and Regional level it would have been expected that more recent strategies would have had a higher linkage score. The results, however, showed that a newer strategies were no more likely to have a higher linkages score than older strategies for both SRHR and HIV. This disconnect appears to be due partly to persistent vertical structures which make linked coordination and planning difficult. Vertical funding structures – especially through poor donor coordination at the international level – and competition for scarce resources between the SRHR and HIV sectors make these national level structures even harder to link despite the inherent logic for doing so.

Eastern and Southern Africa and Western and Central Africa were the regions that had the highest scores for both SRHR and HIV strategies. In these two regions, HIV prevalence is highest, 21 of the 22 highest burden EMTCT countries are located, and there are poorer overall SRH indices (UNAIDS 2015b; WHO *et al*. 2015; UNAIDS 2016; UNFPA 2016). The magnitude of both HIV and SRHR ill-health have necessitated increased need for SRHR and HIV linkages in respective national SRHR and HIV strategies and plans. Regional support for a joint HIV and SRHR response can be seen through the Maputo Plan of Action (African Union 2006) and the minimum standards, such as the SADC *Minimum Standards for the integration of HIV and Sexual and Reproductive Health* (Southern African Development Community 2015).

## Conclusion

The outcome document of ICPD in 1994 specified that SRHR services including HIV should be integrated within a primary healthcare approach and that linked multi-sectoral strategies were an essential component in making this happen. Likewise, since 2004, global HIV commitments and strategies have called for the inclusion of the relevant SRHR components. Whilst there has been a generally increasing commitment over the past 12 years to better link SRHR and HIV, this trend is still not yet resulting in fully connected HIV and SRHR-strategies that provide the vision, targets and ultimately funding for a joint response at the legal/policy, health systems and service delivery levels.

At the legal/policy level, a first step is having SRHR and HIV strategies recognise the key intersections which forms a common understanding and provides a platform for further joint action among the HIV and SRHR communities to change laws and policies to end gender based violence, child marriage and criminalisation of HIV, all forms of stigma and discrimination against PLHIV and key populations.

More comprehensive and resilient health systems require integrated service delivery to be ‘normalised’ rather than being seen as an ‘optional extra’ (Smit *et al.* 2012). Therefore, it is essential that national HIV and SRHR strategies are strengthened to fully incorporate the intersections as measurable targets. Tools such as the *Minimum Standards for the Integration of HIV and Sexual & Reproductive Health in the SADC Region* (Southern African Development Community 2015) are a step in the right direction to support countries to realise joint HIV and SRHR goals starting with linked strategies.

From an HIV perspective, with international donor resources dwindling and a push for increased domestic financing, it is important that key HIV components such as the four prongs of EMTCT, HIV testing and counselling, and antiretroviral treatment (ART) are fully incorporated into SRHR strategies to be taken forward by the Ministry of Health. This is even more important given that some donors and SRHR organisations are beginning to subsume HIV within their SRHR portfolios and organisational units. To advance key elements of the HIV response, including the rights of people living with HIV and key populations, their intersection with each of the SRHR components need to be clearly articulated and promoted.

From an SRHR perspective major donors such as the Global Fund and PEPFAR, are supporting integration of SRHR with HIV and increasingly relying on National Strategic Plans to determine funding priorities (PEPFAR 2011; PEPFAR *et al.* 2015; Global Fund 2016) underscoring the need for national HIV strategies to include SRHR components.

From both the SRHR and HIV perspectives, joint efforts need to be stepped up to better meet the specific SRHR needs of PLHIV. For example family planning programmes need to eliminate coerced/forced sterilisation of women living with HIV, provide the full range of contraceptive options, and be aware of current recommendations regarding possible interactions between hormonal contraception and ART. Conversely ART programmes need to screen for a treat for cervical cancer among women living with HIV, ascertain fertility intentions, provide counselling around choices for a safe pregnancy.

Also, given that condoms confer triple protection against HIV, STIs, and unintended pregnancies, and STIs can increase HIV acquisition and transmission there is a clear argument for more joint work to make the most of these natural synergies. With the UNAIDS targets to increase knowledge of HIV status and access to ART (UNAIDS 2015a), multiple service delivery platforms are required, including for pre- and post-exposure prophylaxis. SIDA, Norad and EU have been funding a multi-country and SRHR and HIV Linkages project in Southern and Eastern Africa, which is providing lessons learned to help guide other efforts to strengthen linkages (UNFPA and UNAIDS 2015).

Bi-directional linkages between SRHR and HIV have numerous benefits but for these to be fully realised, there needs to be a comprehensive approach that focuses equal attention at the legal/policy, health systems and service delivery levels. Improving the extent to which HIV strategies address SRHR and vice versa – an action within the legal/policy level – will help to ‘normalise’ integrated service delivery, reduce duplication and increase the efficiency and resilience of the health system. In this era of challenges to human rights and increasingly limited funding, the best way forward is a linked response from the HIV and SRHR communities – where the areas selected for joint action are informed by the latest evidence.
